# Chemical and engineering bases for green H_2_O_2_ production and related oxidation and ammoximation of olefins and analogues

**DOI:** 10.1093/nsr/nwae243

**Published:** 2024-07-17

**Authors:** Minghua Qiao, Xinggui Zhou, Zexue Du, Peng Wu, Baoning Zong

**Affiliations:** Collaborative Innovation Center of Chemistry for Energy Materials, Department of Chemistry and Shanghai Key Laboratory of Molecular Catalysis and Innovative Materials, Fudan University, Shanghai 200438, China; State Key Laboratory of Chemical Engineering, East China University of Science and Technology, Shanghai 200237, China; State Key Laboratory of Petroleum Molecular & Process Engineering, Research Institute of Petroleum Processing, SINOPEC, Beijing 100083, China; State Key Laboratory of Petroleum Molecular & Process Engineering, School of Chemistry and Molecular Engineering, East China Normal University, Shanghai 200062, China; State Key Laboratory of Petroleum Molecular & Process Engineering, Research Institute of Petroleum Processing, SINOPEC, Beijing 100083, China

**Keywords:** hydrogen peroxide, oxidation, ammoximation, propylene oxide, epichlorohydrin, cyclohexanone oxime

## Abstract

Plastics, fibers and rubber are three mainstream synthetic materials that are essential to our daily lives and contribute significantly to the quality of our lives. The production of the monomers of these synthetic polymers usually involves oxidation or ammoximation reactions of olefins and analogues. However, the utilization of C, O and N atoms in current industrial processes is <80%, which represents the most environmentally polluting processes for the production of basic chemicals. Through innovation and integration of catalytic materials, new reaction pathways, and reaction engineering, the Research Institute of Petroleum Processing, Sinopec Co., Ltd. (RIPP) and its collaborators have developed unique H_2_O_2_-centered oxidation/ammoximation technologies for olefins and analogues, which has resulted in a ¥500 billion emerging industry and driven trillions of ¥s' worth of downstream industries. The chemical and engineering bases of the production technologies mainly involve the integration of slurry-bed reactors and microsphere catalysts to enhance H_2_O_2_ production, H_2_O_2_ propylene/chloropropylene epoxidation for the production of propylene oxide/epichlorohydrin, and integration of H_2_O_2_ cyclohexanone ammoximation and membrane separation to innovate the caprolactam production process. This review briefly summarizes the whole process from the acquisition of scientific knowledge to the formation of an industrial production technology by RIPP. Moreover, the scientific frontiers of H_2_O_2_ production and related oxidation/ammoximation processes of olefins and analogues are reviewed, and new technological growth points are envisaged, with the aim of maintaining China's standing as a leader in the development of the science and technologies of H_2_O_2_ production and utilization.

## Teaser

This review overviews China’s unique green chemical technologies for H_2_O_2_ production and hydrocarbon oxidation/ammoximation with the global market share exceeding 60%.

## INTRODUCTION

Using petroleum-based hydrocarbons to fabricate O- or N-containing organic compounds by means of oxidation or ammoximation is an important way to obtain basic organic chemicals, organic synthesis intermediates and fine chemicals, and occupies an extremely important position in modern chemical industry [1]. More than 50% of the chemicals are fabricated by involving the oxidation reaction. And the fabrication of key monomers for synthetic fibers, resins, rubbers, drugs, pesticides and fine chemicals involves oxidation or ammoximation. The as-fabricated chemicals and materials are not only widely used in daily life, such as in electronics, electricity, transportation, machinery manufacturing, medical treatment and agriculture, but also have important applications in the fields of national defense and aerospace.

However, industrial hydrocarbon oxidation processes mostly use highly toxic reagents, e.g. dichromate and permanganate, with hypochlorite and nitric acid as the oxidants. To obtain the target O-containing organic compounds, stoichiometric amounts of inorganic wastes or NO*_x_* are generated, causing poor atomic economy and severe environmental pollution. On the other hand, hydrocarbon ammoximation uses nitric acid, hydroxylamine, azide or highly toxic cyanide as the active nitrogen reagent. Nitric acid is synthesized by NH_3_ oxidation, which emits more than 300 kt of NO*_x_* per year, accounting for 30% of NO*_x_* emissions from the chemical industry [2]. Hydroxylamine is produced by NO_3_^–^ reduction, which involves NH_3_ oxidation, SO_2_ reduction and NO_3_^–^ reduction, and generates (NH_4_)_2_SO_4_ by-product and NO*_x_* and SO*_x_* emissions. Moreover, the production of nitric acid and hydroxylamine is energy intensive and emits a large amount of CO_2_ [3]. When using these active nitrogen reagents to fabricate the nitrogen-containing organic compounds, salt wastes are also generated, leading to <60% N utilization.

**Figure 1.**
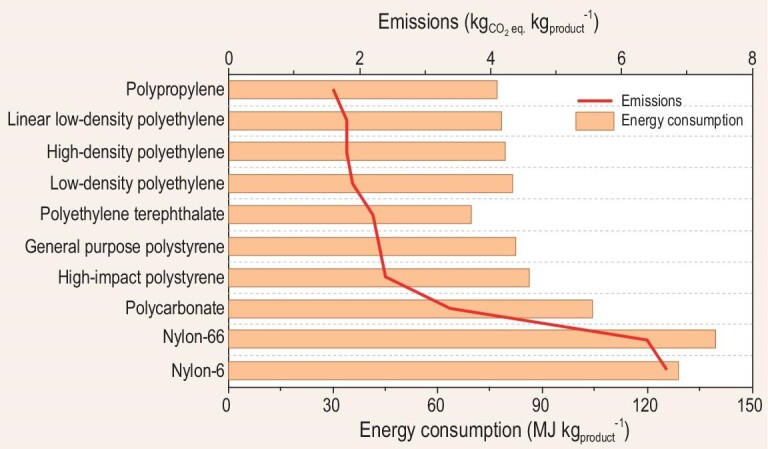
Energy consumption and equivalent carbon emissions per unit weight of polymers [4].

Based on the life cycle inventory theory, the European Plastics Manufacturers Association converted NO*_x_* into greenhouse gas CO_2_ equivalent, and calculated the environmental impact of the whole production process of various polymers [4]. The energy consumption and carbon emissions for the production of a unit weight of the polymers are illustrated in Fig. 1. The energy consumption and carbon emissions per unit weight of nylon-6 and nylon-66 are much higher due to multiple oxidation and ammoximation steps. For example, only a single adipic acid production step accounts for 10% of the incremental global N_2_O emissions. Therefore, it is essential to develop novel green reaction technologies to reduce energy consumption and NO*_x_* emissions and to improve the atomic economy of the industrial oxidation and ammoximation processes.

To this end, the choice of active oxygen and nitrogen reagents is crucial. The by-product of H_2_O_2_, as a green and strong oxidant, is only water, which is not only widely used in hydrocarbon oxidation, but has also attracted attention with regard to the one-step synthesis of N-containing organic compounds by ammoximation [5]. In 2018, the domestic consumption of H_2_O_2_ exceeded 3.2 Mt (100% basis), accounting for >50% of global H_2_O_2_ consumption [6], and is still growing at a rate of >5% annually. However, the fixed-bed technology is still used for the production of H_2_O_2_ in China, which has shortcomings: low single-unit productivity and high production cost.

To accelerate the green transformation of the chemical industry in China, RIPP has successfully developed slurry-bed technology for H_2_O_2_ production after more than two decades of work. Meanwhile, a number of green production technologies for the fabrication of basic organic chemicals including propylene oxide (PO), epichlorohydrin (ECH) and cyclohexanone oxime (CHO) by hydrocarbon oxidation or ammoximation using H_2_O_2_ as the oxidant have been developed and applied in many world-class chemical production bases.

This review gives a brief introduction to the current technologies for the production of H_2_O_2_, PO, ECH and CHO, with special emphasis on the contributions of RIPP and its collaborators to the development of green production technologies for the above processes. Furthermore, progress in H_2_O_2_ production and related oxidation and ammoximation for PO, ECH and CHO production is addressed. Finally, a summary and outlook on the scientific challenges and opportunities of these processes are provided.

## PRODUCTION TECHNOLOGY OF H_2_O_2_

### Current status

**Figure 2.**
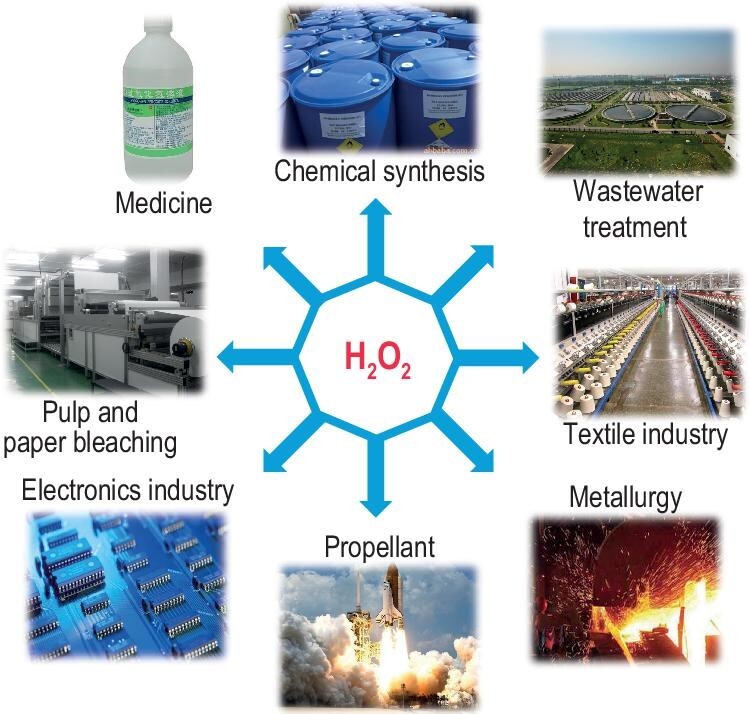
Principal uses of H_2_O_2_.

H_2_O_2_ is widely used in fields such as medical sterilization, wastewater treatment, papermaking, textile, aerospace, metallurgy and electronics (Fig. 2). The content of active oxygen in H_2_O_2_ is as high as

**Figure 3.**
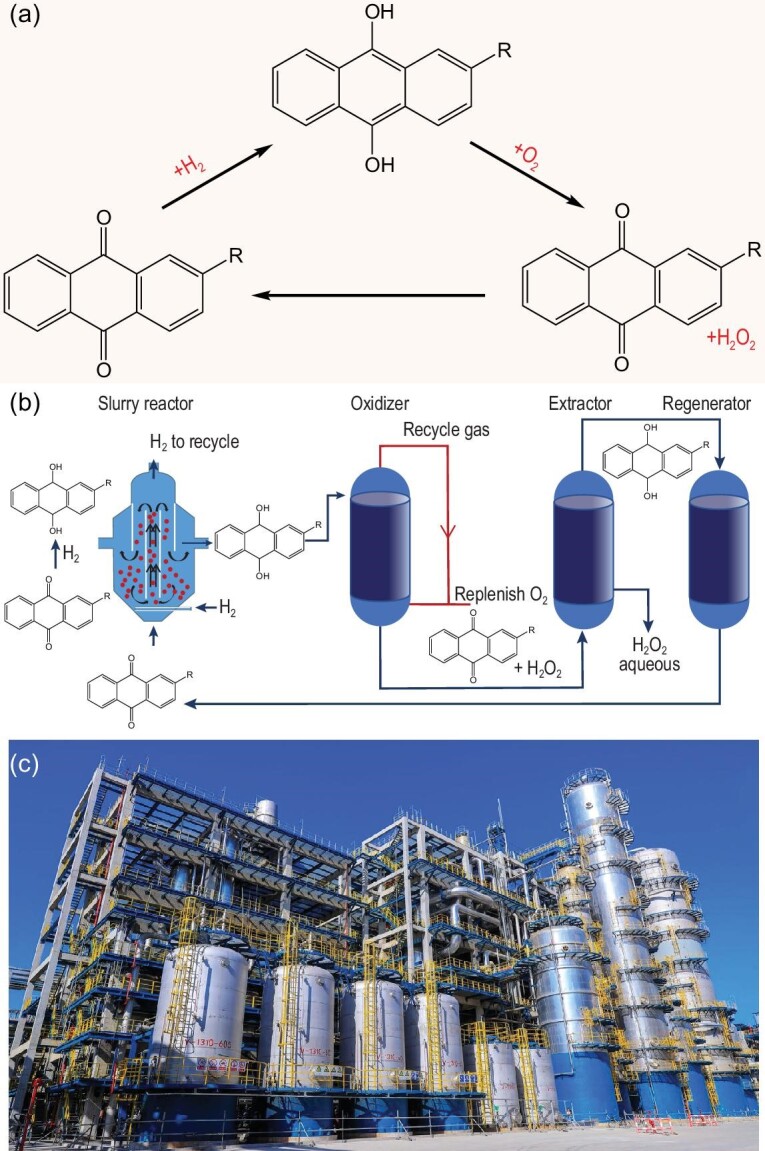
(a) The main reactions for the production of H_2_O_2_ via the anthraquinone process. (b) Schematic diagram of the slurry-bed technology for the production of H_2_O_2_ developed by RIPP. (c) The 240 kt a^−1^ H_2_O_2_ production unit based on RIPP's slurry-bed technology.

47.1 wt%. Currently, >95% of the market share of H_2_O_2_ is produced by the anthraquinone process (Fig. 3a) due to high catalytic efficiency and technical maturity [6,7]. The anthraquinone process in China is mainly based on fixed-bed technology. Since the heat and mass transfer of the fixed-bed reactor is poor, excessive hydrogenation and hence the degradation of the expensive alkylanthraquinone will occur, thus giving a low hydrogenation efficiency of 7.0–7.5 g L^−1^ [8]. An acid–base environment switch is required in successive processes, which increases the operation risk and environmental pollution. Moreover, the production capacity of a fixed-bed-technology-based plant is limited to 50 kt a^−1^ due to poor heat and mass transfer.

In contrast, slurry-bed technology for anthraquinone hydrogenation is advantageous in heat and mass transfer. Therefore, the temperature distribution is more uniform, which renders higher hydrogenation efficiency and larger production scales. The working solution degradation and catalyst consumption can be substantially reduced [9]. The production capacity of a slurry-bed-technology-based plant is generally larger than 100 kt a^−1^. The H_2_O_2_ concentration in the raffinate is below 0.05 g L^−1^, and the slurry-bed process is operated in a fully acidic environment, thus guaranteeing intrinsic operation safety. However, the hydrogenation catalyst used in the slurry-bed process demands high strength in addition to high selectivity. RIPP systematically investigated support modification, active metal loading and catalyst shaping, and improved the hydrogenation efficiency by 1.5 times and decreased the wear index by half. The spherical support and the Pd-loaded egg-shell catalyst were uniform in shape and had stable physicochemical properties, which, in combination with the synergistic effect of the metal promoter, significantly improved the hydrogenation activity. Industrial testing showed that the hydrogenation efficiency reached 12–13 g L^−1^ without obvious changes in the particle size and structure of the catalyst [10].

The working solution is one of the main factors affecting hydrogenation efficiency and productivity. As the core component of the working solution, 2-alkylanthraquinone is traditionally synthesized by the highly polluting phthalic anhydride method. In contrast, the synthesis of 2-alkylanthraquinone by 2-alkylanthracene oxidation is green. By using Y-type zeolite as the catalyst and 1,3,5-trimethylbenzene as the solvent, RIPP improved the selectivity to 2-alkylanthraquinone in a membrane reactor and increased the purity by melt crystallization and multi-stage vacuum distillation. In addition, by optimizing the ratio of tetrahydro-2-amylanthraquinone to 2-amylanthraquinone, the hydrogenation efficiency was increased by at least 30% [10]. Degradation of the working solution reduces the productivity of H_2_O_2_ and increases anthraquinone consumption. By determining the identities of the degradation products [11], RIPP developed a regeneration technology by catalytically oxidizing anthrone back to effective anthraquinone, which improved the conversion to effective anthraquinone 10-fold.

Air is traditionally used to oxidize hydroalkylanthraquinones, which results in >98% of total tail gas emissions in H_2_O_2_ production [12]. The high-boiling-point aromatics in the tail gas are not only hazardous, but also induce great safety risks. Moreover, the condensation and accumulation of the compressed-air-entrained moisture is the main source of residual liquid in the oxidation tower. It was estimated that the condensate generation rate was as high as 120.3 kg h^−1^ at 30°C for a 45 kt a^−1^ H_2_O_2_ plant [6]. By means of pressurized cycling of the oxidation tail gas and quantitatively replenishing pure O_2_, RIPP eliminated tail gas emissions in the oxidation step.

In light of these improvements, a slurry-bed process for H_2_O_2_ production has been successfully demonstrated by RIPP. Figure 3b schematically illustrates the general principle of the RIPP's slurry-bed technology. The slurry containing the working solution and the sphere catalyst enters the slurry-bed reactor from the bottom. Buoyed by H_2_, the microsphere catalyst is in full contact with the working solution and H_2_ in the reaction zone. H_2_ is adsorbed and dissociated on the microsphere catalyst, which reduces 2-amylanthraquinones diffused onto the microsphere catalyst to hydroanthraquinones. The hydrogenation heat and hydroanthraquinones can be more quickly released into the reaction solution using the slurry-bed reactor than using the traditional fixed-bed reactor, thus avoiding local overheating and excessive hydrogenation of hydroanthraquinones. As a result, the hydrogenation degree of 2-amylanthraquinones can be as high as 70% on the slurry-bed reactor as compared to 30%–40% on the fixed-bed reactor to avoid excessive hydrogenation. The slurry containing the working solution and the microsphere catalyst flow from the top to the subsidence zone, where the separation of the microsphere catalyst and the working solution is accomplished. The microsphere catalyst forms slurry at the bottom of the subsidence zone, which is buoyed by H_2_ again and recirculated into the reaction zone. The working solution flows out from the side outlet of the slurry-bed reactor and enters the oxidation tower. The oxidation gas is prepared by quantitative replenishment of pure O_2_ into the circulating oxidation tail gas to eliminate tail gas emissions. The oxidized solution enters the extraction column to extract H_2_O_2_. The regenerated working solution is returned to the slurry-bed reactor. Since the microsphere catalyst is in the flow state in the slurry-bed reactor, the constant collision and friction will cause the catalyst to fracture, so it puts higher requirements on the strength and surface wear resistance of the microsphere catalyst. The excellent heat and mass transfer makes the slurry-bed technology easier to scale up, and the H_2_O_2_ production capacity on a single unit can reach 240 kt a^−1^, which satisfies the needs of 300 kt a^−1^ PO, ECH or cyclohexanone oxime devices. As compared to the process based on fixed-bed technology (Table 1), the production capacity of a single unit using slurry-bed technology increases 2-fold. The energy and material consumptions are reduced by ∼20%. There are no tail gas emissions. The wastewater discharge is reduced by 30%. The operation process is in a fully acidic mode with high intrinsic safety [6]. Recently, a 240 kt a^−1^ H_2_O_2_ production unit based on this technology was successfully established (Fig. 3c).

**Table 1.** Comparison of the main technical parameters of anthraquinone processes based on the slurry-bed reactor and fixed-bed reactor.

Process	Hydrogenation efficiency (g L^−1^)	Working solution loss (kg t_H_2_O_2__^−1^)	Energy consumption (kWh t_H_2_O_2__^−1^)	Waste gas (m^3^ t_H_2_O_2__^−1^)	Waste water (m^3^ t_H_2_O_2__^−1^)	Max. capacity per unit (kt a^−1^)	H_2_O_2_ cost (¥ t^−1^)
Slurry bed	12–13	2.0	580	0	0.11	240	2100
Fixed bed	7–7.5	2.5	733	1387	0.15	50	2283

### Alternative H_2_O_2_ synthesis processes

#### H_2_–O_2_ direct synthesis

**Scheme 1.**
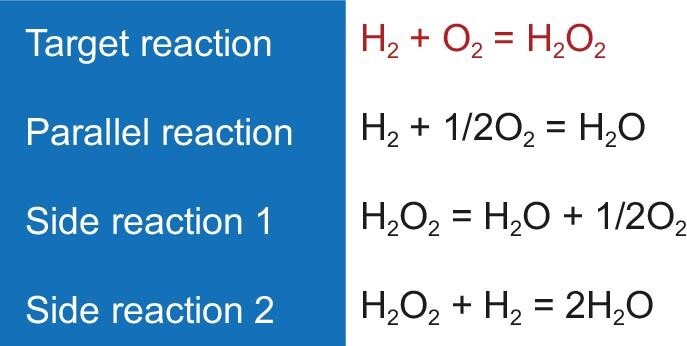
H_2_–O_2_ direct synthesis of H_2_O_2_ and parallel and side reactions.

H_2_–O_2_ direct synthesis is the most direct method of H_2_O_2_ production (Scheme 1), and has the advantages of higher atomic economy and lower feedstock and operation costs than the anthraquinone process [13,14]. However, during H_2_–O_2_ direct synthesis, H_2_O is inclined to be formed. Meanwhile, H_2_O_2_ tends to decompose into H_2_O and O_2_ or be further reduced to H_2_O. These reactions are all thermodynamically favored, so how to improve the selectivity to H_2_O_2_ is a challenging task.

It was confirmed that the non-dissociative adsorption of O_2_ on Pd is essential for H_2_–O_2_ direct synthesis of H_2_O_2_, and the Pd(111) surface favors the formation of H_2_O_2_, since the adsorption energies of O_2_ and H_2_O_2_ on the coordinatively saturated sites are weaker than on the coordinatively unsaturated corner and edge sites [15]. Alloying Pd with Au, Te, Pt, Ru, Ag or Sn [16] can improve the selectivity to H_2_O_2_ by means of the geometric effect or electron transfer. Over the Pd–Au bimetallic catalyst, H_2_O_2_ selectivity was improved to 98% by Au blocking of the active sites for H_2_O_2_ hydrogenation [13] or weakening the interaction between Pd and H_2_O_2_. Alloying with Te improved H_2_O_2_ selectivity to close to 100% by inhibiting the dissociative adsorption of O_2_ on Pd. Freakley *et al.* [17] prepared a bimetallic Pd-Sn catalyst, on which the H_2_O_2_ selectivity was higher than 95%, and the hydrogenation of H_2_O_2_ was suppressed. Two forms of Sn were identified. One is alloyed with Pd, which enhances activity, and the other is in the form of SnO_2_ film covering small Pd particles, which inhibits side reactions.

H_2_O_2_ is more stable in acidic conditions, so liquid acid is often added to the reaction medium to inhibit the decomposition of H_2_O_2_, or halide is added to poison the catalyst to inhibit the formation of H_2_O [16]. Since liquid acid and halide are highly corrosive, it is ideal if acidic support can play a similar role. Blanco-Brieva *et al*. [18] deposited Pd nanoparticles (NPs) on sulfonic-acid-functionalized SiO_2_, on which high-concentration H_2_O_2_ solution was produced.

Water is frequently used as the solvent for H_2_–O_2_ direct synthesis. However, the solubilities of H_2_ and O_2_ in water are very low, which slows down the formation of H_2_O_2_. H_2_ and O_2_ are miscible with liquid or supercritical CO_2_ in any ratio. Even under subcritical conditions, the solubilities of H_2_ and O_2_ in CO_2_ are much higher than in organic solvents or water, making CO_2_ an ideal solvent for H_2_–O_2_ direct synthesis. Supercritical conditions are preferred to eliminate the gas–liquid mass transfer limitation. In addition, the solubility of H_2_O_2_ in CO_2_ is very low. Thus, once H_2_O_2_ is formed, it is expelled from the solvent to form a bi-phase system. Hâncu and Beckman [19] prepared a Pd catalyst with ligands miscible with CO_2_ at moderate pressures, which showed good performance in H_2_–O_2_ direct synthesis of H_2_O_2_.

Direct synthesis of H_2_O_2_ from H_2_ and O_2_ confronts the following drawbacks. The explosion range of H_2_ in O_2_ is broad (4.0%–95% by volume), so the H_2_/O_2_ ratio should be strictly controlled, or inert gas is added as a diluent. The presence of a large amount of diluent gas greatly reduces H_2_O_2_ production efficiency. As to the selectivity, the catalysts good at producing H_2_O_2_ are in general reactive for H_2_ combustion and H_2_O_2_ decomposition. Therefore, this method is still in need of substantial breakthroughs in catalyst design and reaction engineering.

#### Photocatalytic synthesis of H_2_O_2_

**Figure 4.**
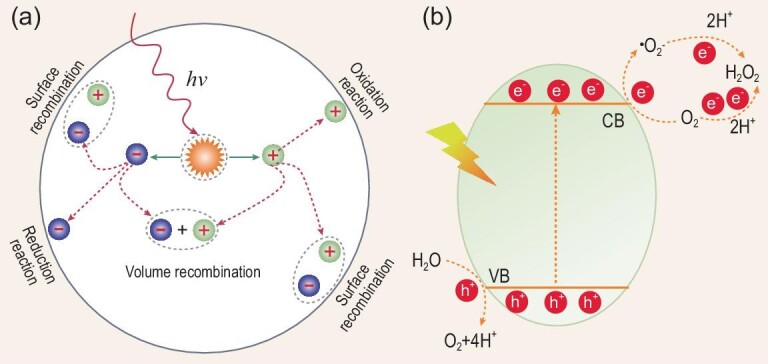
(a) Photoexcitation and the charge-decay pathway in a photocatalyst. (b) Schematic of the photocatalytic system for the production of H_2_O_2_, reproduced with permission from ref. [21]. Copyright 2020, John Wiley and Sons.

The conversion of solar energy into H_2_O_2_ by photocatalysis can direct the conversion and stable storage of solar energy into value-added chemicals. The photocatalytic synthesis methods of H_2_O_2_ include photocatalytic O_2_ reduction and photocatalytic H_2_O oxidation [20]. Photocatalytic synthesis of H_2_O_2_ is devoid of the explosion risk and occurs at ambient temperature and pressure. Due to the low catalytic efficiency, photocatalytic H_2_O oxidation to H_2_O_2_ is rarely reported. Photocatalytic O_2_ reduction to H_2_O_2_ occurs in a two-step one-electron reduction pathway or a one-step two-electron reduction pathway after photo-excitation of the hole (h^+^)–electron (e^–^) pairs (Fig. 4) [21].

The catalysts for photocatalytic O_2_ reduction to H_2_O_2_ can be divided into metal oxide/sulfide semiconductors and organic polymer semiconductors. Metal oxide catalysts mainly include metal oxide semiconductors and metal- or graphene-doped metal oxides. TiO_2_ is the most commonly used metal oxide photocatalyst for H_2_O_2_ synthesis [22]. However, TiO_2_ has poor light absorption ability due to its wide band gap [21]. The concentration of H_2_O_2_ generated on TiO_2_ is very low (<0.2 mM), since H_2_O_2_ can immediately react with the catalyst surface to form the Ti–OOH species. Noble metal modification of TiO_2_ is an effective approach to enhance the photocatalytic activity. UV irradiation of the noble metal/TiO_2_ photocatalysts in O_2_-saturated ethanol/water mixtures produced H_2_O_2_ at the 10 mM level [23]. The promotion mechanism is that first, due to the formation of the Schottky barrier at the noble metal/TiO_2_ interface, the electrons in the TiO_2_ conduction band are readily trapped by the noble metal particles, so the photo-excited charge carriers are effectively separated. The formation of the Schottky barriers also inhibits the formation of the Ti–OOH species. Second, when the noble metal particles are highly uniformly dispersed, visible light can be absorbed by the surface plasmon resonance (SPR) effect.

Hirakawa *et al*. [24] reported that over the Au_0.2_/BiVO_4_ catalyst, the concentration of H_2_O_2_ reached 40.2 μM under visible light irradiation. In particular, this catalyst did not require ethanol as the sacrificial agent, which greatly reduced the production cost. Isaka *et al*. [25] demonstrated that the deposition of the NiO NPs on MIL-125-NH_2_ accelerated the formation of H_2_O_2_. They further developed a bi-phase system consisting of benzyl alcohol and water using hydrophobic metal-organic framework (MOF) as the photocatalyst, which allowed the formation of H_2_O_2_ in the aqueous phase while eliminating the separation step from benzaldehyde [26].

Organic polymer semiconductors for photocatalytic H_2_O_2_ synthesis are mainly based on graphitic phase carbon nitride (*g*-C_3_N_4_) [27]. *g*-C_3_N_4_, with a band gap of ∼2.7 eV corresponding to the wavelength of ca. 460 nm, is a potential photocatalyst responsive to visible light [28]. However, the photocatalytic activity of *g*-C_3_N_4_ is low due to low specific surface area, poor visible light absorption and fast recombination of the charge carriers. Morphology adjustment, defect engineering, noble metal loading, doping and organic structural unit functionalization can increase the active sites [29], narrow down the band gap [30], improve the density of the charge carriers [31], accelerate the separation and transport of charge carriers [32,33], and improve O_2_ adsorption [32,34], thereby improving the photocatalytic activity of *g*-C_3_N_4_.

Tian *et al*. [35] synthesized a K and P-doped carbon nitride photocatalyst, on which the H_2_O_2_ concentration exceeded 5 mM after 10 h of reaction, which is more than 5-fold that on *g*-C_3_N_4_ synthesized from urea. Qu *et al*. [36] synthesized alkali metal (K^+^, Na^+^) doped *g*-C_3_N_4_ (MCN). Under visible light irradiation, the equilibrium concentration of H_2_O_2_ was 4.9 mM, which was a 9-fold increase in activity relative to that of *g*-C_3_N_4_. Zhang *et al*. [37] calcined a mixture of KOH and KCl with melamine to synthesize AKCN, and 3.4 mM of H_2_O_2_ was produced under visible light irradiation for 3 h. They subsequently synthesized AKMT by calcining a mixture of NaOH, KCl, thiourea and melamine, on which 4.1 mM of H_2_O_2_ was produced [32]. The K and S dopants in the AKMT promoted the separation of internal charges. The polarization of *g*-C_3_N_4_ in the presence of the K and S dopants favored the capture of electrons for reacting with O_2_. Li *et al*. [38] synthesized a K, S and O co-doped PCN (*akut*-CN) via polymerization of urea and thiourea in the presence of KCl and NaOH, which afforded an unprecedentedly high H_2_O_2_ production rate of 4.46 mM h^−1^ under visible light irradiation. The heteroatom doping and the incorporation of the cyano and hydroxyl groups were proposed to enhance light absorption, accelerate the separation and transfer of the charge carriers, and improve the O_2_ adsorption capacity and strength, which collaboratively boosted the reaction kinetics.

Photocatalytic reduction of O_2_ to H_2_O_2_ completely avoids the explosion risk. However, the activity of the photocatalysts needs substantial improvement to satisfy the demands of practical applications [39], as the production efficiency of H_2_O_2_ for the time being is only about one-tenth of that of H_2_–O_2_ direct synthesis.

### Electrocatalytic reduction of O_2_ to H_2_O_2_

Electrocatalytic two-electron reduction of O_2_ (2e-ORR) is an ideal method for H_2_O_2_ production. In aqueous solution, the cathodic O_2_ reduction reaction (ORR) can occur via two pathways, i.e. 2e-ORR and four-electron ORR (4e-ORR) [40]. In acidic electrolytes, the reactions are:


(1) \begin{eqnarray*}
{{{\mathrm{O}}}_2} + 2{{{\mathrm{H}}}^ + } + 2{{{\mathrm{e}}}^-} = {{{\mathrm{H}}}_2}{{{\mathrm{O}}}_2},\quad{{E}^0} = 0.70\,{\mathrm{V}}\,{\mathrm{vs}}.\nonumber\\ {\mathrm{reversible\ hydrogen\ electrode\ (RHE)}};
\end{eqnarray*}

(2) \begin{equation*}
{{{\mathrm{O}}}_2} + 4{{{\mathrm{H}}}^ + } + 4{{{\mathrm{e}}}^-} = 2{{{\mathrm{H}}}_2}{\mathrm{O}},\quad{{{{E}}}^0} = 1.23\,{\mathrm{V}}\,{\mathrm{vs}}.\,{\mathrm{RHE}}.
\end{equation*}

In basic electrolytes, the reactions are:


(1′) \begin{eqnarray*}
&&{{{\mathrm{O}}}_2} + 2{{{\mathrm{H}}}_2}{\mathrm{O}} + 2{{{\mathrm{e}}}^-} = {{{\mathrm{H}}}_2}{{{\mathrm{O}}}_2} + 2{\mathrm{O}}{{{\mathrm{H}}}^-},\nonumber\\
&&\quad \quad \quad {{{{E}}}^0} = 0.70\,{\mathrm{V}}\,{\mathrm{vs}}.\,{\mathrm{RHE}};
\end{eqnarray*}

(2′) \begin{eqnarray*}
&&{{{\mathrm{O}}}_2} + 4\,{{{\mathrm{H}}}_2}{\mathrm{O}} + 4\,{{{\mathrm{e}}}^-} = 4{\mathrm{O}}{{{\mathrm{H}}}^-},\nonumber\\
&&\quad \quad \quad \quad {{{\mathrm{E}}}^0} = 1.23\,{\mathrm{V}}\,{\mathrm{vs}}.\,{\mathrm{RHE}}.
\end{eqnarray*}

At pH>11.7, the 2e-ORR becomes:


(1′′) \begin{eqnarray*}
&&{{{\mathrm{O}}}_2} + {{{\mathrm{H}}}_2}{\mathrm{O}} + 2{{{\mathrm{e}}}^-} = {\mathrm{H}}{{{\mathrm{O}}}_2}^- + 2{\mathrm{O}}{{{\mathrm{H}}}^-},\nonumber\\
&&\quad\quad\quad\quad {{{{E}}}^0} = 0.76\,{\mathrm{V}}\,{\mathrm{vs}}.\,{\mathrm{RHE}}.
\end{eqnarray*}

The as-produced H_2_O_2_ can be further reduced to H_2_O, thus completing the 4e-ORR pathway:


(3) \begin{eqnarray*}
&&{{{\mathrm{H}}}_2}{{{\mathrm{O}}}_2} + 2{{{\mathrm{H}}}^ + } + 2{{{\mathrm{e}}}^-} = 2{{{\mathrm{H}}}_2}{\mathrm{O}},\nonumber\\
&&\quad \quad \quad {{E}^0} = 1.76\,{\mathrm{V}}\,{\mathrm{vs}}.\,{\mathrm{RHE}}.
\end{eqnarray*}

In the 1980s, Dow and Huron developed a trickle-bed electrochemical reactor for the production of H_2_O_2_ by O_2_ cathodic reduction, known as the Dow-Huron process [41]. Using graphite flakes coated with a mixture of carbon black and polytetrafluoroethylene (PTFE) as the cathode, 2 wt% of H_2_O_2_ was produced in a NaOH solution at 25°C, 2 V and 0.031 A cm^–2^. The main drawbacks of the Dow-Huron process are the high basicity of the electrolyte, in which H_2_O_2_ tends to decompose. Moreover, the electrolyte causes corrosion, and the ceramic membrane separating the anode and cathode results in high resistance.

Yamanaka *et al*. [42] designed a device fixed with a porous carbon composite cathode fabricated by hot-pressing carbon fiber with PTFE and using NaOH as the electrolyte. This device solved the problem of low O_2_ solubility and increased the O_2_ concentration on the porous cathode, which not only accelerated the reduction of O_2_ to H_2_O_2_, but also inhibited H_2_O_2_ from further reduction to H_2_O. As a result, the H_2_O_2_ concentration and selectivity reached 7 wt% and 93%, respectively. Later on, Yamanaka and Murayama [43] improved the device by using a solid electrolyte, and obtained a neutral 8 wt% H_2_O_2_ solution.

Johnson-Matthey investigated the synthesis of H_2_O_2_ in an H_2_/O_2_ fuel cell under acidic conditions. Loading 2 wt% of Co onto activated carbon as the electrode allowed the synthesis of 30 wt% H_2_O_2_. The device was continuously operated in the bench scale for several months without significant deactivation [44].

**Figure 5.**
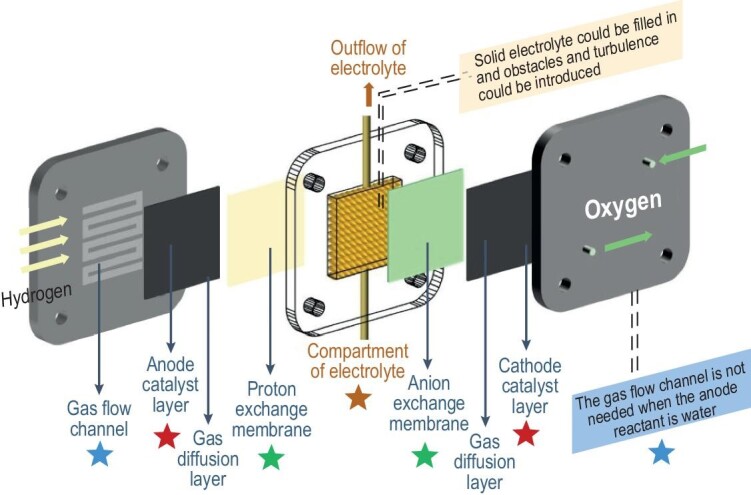
The components and key operating principles of devices for H_2_O_2_ electrosynthesis, reproduced with permission from ref. [46]. Copyright 2022, John Wiley and Sons.

Xia *et al*. [45] developed a solid electrolyte fuel cell with H_2_/H_2_O and O_2_ separately flowing to the anode and cathode, respectively. A proton-exchange membrane (PEM) and an anion-exchange membrane (AEM) were used between the middle chamber and the electrodes to avoid the electrodes being immersed by water [46]. In this way, HO_2_^–^ from the cathode and H^+^ from the anode were transported to the middle chamber, where they combined as H_2_O_2_ with the promotion of the solid electrolyte layer, which was collected with deionized water (Fig. 5). As such, H_2_O_2_ aqueous solutions with a wide range of concentrations could be obtained by adjusting the flow rate of deionized water without introducing ionic impurities. Up to 20 wt% of H_2_O_2_ aqueous solution could be obtained on this device.

To enhance electrochemical O_2_ reduction to H_2_O_2_, much research is focused on 2e-ORR at the cathode. Similar to that of the H_2_/O_2_ fuel cell, the electrolyte is acidic, alkaline or neutral. The catalysts are noble metals, carbon materials and carbon materials loaded with noble metals. Noble metal catalysts are mainly used in acidic systems, and the selectivity to H_2_O_2_ is generally higher than 90%, but the high cost of the noble metals limits their practical application.

The carbon-based catalysts are ideal electrocatalysts for 2e-ORR due to high resistance to acid and alkali corrosion, excellent electrical conductivity and low cost [47]. The main drawback is their weak interaction with the intermediates formed during 2e-ORR, which causes low activity. The 2e-ORR activity and H_2_O_2_ selectivity can be improved by tuning the morphology, composition and electronic structure of the carbon materials [48]. Besides, doping is an effective strategy to improve the 2e-ORR catalytic performance of the carbon-based catalysts. Heteroatoms such as N, O, F and B have been doped or co-doped into the carbon-based materials [49–60]. Due to the discrepancy in the electronegativity between the carbon atom and the heteroatom, the integrity of the π-conjugated system is disrupted, which results in the redistribution of the charge and variation in the adsorption behavior of the reaction intermediate *OOH [40]. Moreover, heteroatom doping can introduce additional functional groups onto the carbonaceous skeleton, which may serve as the active sites.

For the N-doped carbon-based catalysts, pyrrolic N [59], a combination of pyridinic N and pyrrolic N [60], or graphitic N [61] favors H_2_O_2_ production. Sun *et al*. [61] suggested that pyridinic N is the active site in acidic solution, while graphitic N is more likely the active site in neutral and alkaline solutions. For the O-doped catalysts, COOH, C=O [56], C–O–C [58] and the quinone group [55] have been proposed to show better 2e-ORR activity. The incorporation of F disrupted the charge homogeneity of the carbon material, and the CF_2_ and CF_3_ species favored the adsorption of O_2_ and facilitated the desorption of the *OOH intermediate. This is beneficial to the production of H_2_O_2_ [54]. For the N and O co-doped catalysts, the synergistic effect of C–N/C–COOH reduced the overpotential of the 2e-ORR reaction [51,52]. For the N and F co-doped carbon cages, N promoted the adsorption of O_2_, and F promoted the desorption of the *OOH intermediate [50]. For B and N co-doped carbon materials, *h*-BN domains were identified, and 2e-ORR preferentially occurred at the interface between the *h*-BN domain and the graphene lattice [49].

Electrocatalytic reduction of O_2_ to H_2_O_2_ is greener and more economic than the anthroquinone method and far more efficient than the photocatalytic process. Its catalytic efficiency is comparable to or higher than H_2_–O_2_ direct synthesis while devoid of the explosion risk, making it the method with the most potential to replace the anthroquinone method.

As an alternative to electrocatalytic reduction of O_2_ to H_2_O_2_, the two-electron water oxidation reaction (2e-WOR, 2H_2_O = H_2_O_2_ + 2H^+^ + 2e^–^, *E*^0^ = 1.76 V vs. RHE) can also be utilized to produce H_2_O_2_. However, the 2e-WOR is not as competitive as 2e-ORR, as it is thermodynamically less favorable than the oxygen evolution reaction (OER, 2H_2_O = O_2_ + 4H^+^ + 4e^–^, *E*^0^ = 1.23 V vs. RHE), and H_2_O_2_ tends to be oxidized or disproportionated at the potential for 2e-WOR. Moreover, for the time being, most of the catalysts proposed for 2e-WOR are unable to operate at the high current densities (>100 mA cm^–2^) required for the large-scale implementation of the 2e-WOR. Although boron-doped diamond (BDD) microfilms displayed attractive activity and stability for 2e-WOR, to advance the large-scale implementation of BDD, a better understanding of the exact influence of various diamond film properties on H_2_O_2_ selectivity and productivity is required [62].

## PROPYLENE OXIDATION TO PROPYLENE OXIDE

### Current status

**Scheme 2.**
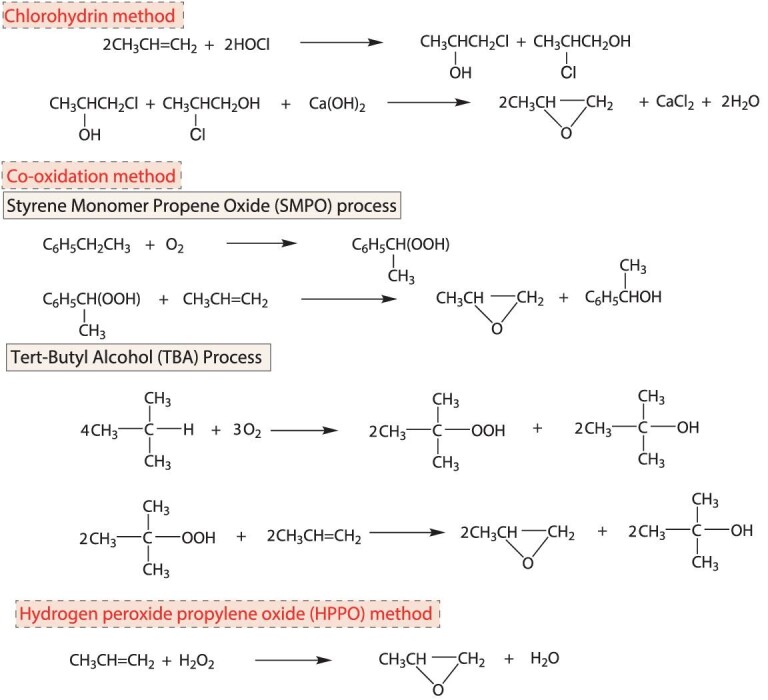
The main industrial methods for the production of PO.

PO is the second largest propylene derivative. About 65% of the total PO consumption is for the synthesis of polyether polyols to fabricate polyurethane foams, and ∼20% is for the production of propylene glycol to fabricate unsaturated polyester resins, surfactants, tobacco wetting agents, plasticizers and cosmetic solvents. PO is also used to synthesize chemicals such as propylene carbonate and alcohol ethers. These products have wide applications in the automotive industry, textiles, construction, agriculture, cosmetics and food. In 2020, the output of PO in China was 2900 kt, while the output was ∼10 500 kt worldwide. The main industrial production processes of PO are the chlorohydrin method, co-oxidation method and hydrogen peroxide propylene oxide (HPPO) method (Scheme 2) [63]. At present, ∼40% of the total PO production capacity in China is based on the chlorohydrin method, 43% on the co-oxidation method, and 17% on the HPPO method.

The chlorohydrin method uses hypochlorous acid as the oxidant. The reaction involves Cl_2_ and causes severe equipment corrosion. Moreover, for the production of 1 t of PO, 1.35–1.85 t of Cl_2_ is consumed, while 40–80 t of Cl-containing wastewater and more than 2 t of CaCl_2_ are produced. The co-oxidation method has the disadvantages of having a complex process, harsh reaction conditions that demand high-quality equipment material, and consumption of H_2_. The economic benefits of the co-oxidation method using ethylbenzene or *iso*-butane as co-oxidizer are constrained by the co-products, as 2.2–2.5 t of styrene or 2.3 t of *tert*-butanol are produced when producing 1 t of PO, which means an atom utilization of only 30%. The technical route with cumene as the co-oxidizer has no co-product, but the separation and conversion of the intermediate products increase energy consumption.

The HPPO method uses H_2_O_2_ as the oxidant and titanium silicate (TS) zeolite as the catalyst for direct epoxidation of propylene to PO. The by-product is only water, and no corrosive reagent is used. As compared to traditional PO production methods, the carbon atom utilization of the HPPO method is close to 100%, and the plant investment, wastewater discharge and energy consumption can be reduced by 25%, 70%–80% and >35%, respectively, rendering HPPO a green method for PO production. However, the HPPO method is technically challenging and was previously mastered only by BASF-Dow and Evonik.

RIPP found that the acidity of the TS zeolite is the main factor that accelerates the solvolysis of PO, and the acidity originates from the Ti active centers, trace Al and surface defects. Therefore, a surface silicon-rich hollow TS zeolite with abundant mesopores and enhanced pore volume was synthesized. RIPP also developed an *in-situ* catalyst regeneration technology with solvent extraction. The catalytic performance of the regenerated catalyst was comparable to that of the fresh catalyst with a lifetime of up to 2000 h. Since the HPPO reaction is strongly exothermic, temperature control is essential for safe operation. By adopting a two-stage fixed-bed reactor in series, RIPP not only overcame the shortcomings of low H_2_O_2_ conversion and poor PO selectivity in a single reactor, but also realized the continuous production of PO with the aid of *in-situ* catalyst regeneration.

The above improvements, along with the design and manufacture technology of large-scale tubular reactors and the safety control technology, make RIPP one of only three licensors of the HPPO process in the world. The 1 kt a^−1^ pilot-scale demonstration enabled a H_2_O_2_ conversion of 96%–99% and a PO selectivity of 96%–98%. There was no significant change in the catalytic activity after 6000 h of reaction. The purity of PO after double azeotrope distillation was no less than 99.97% [64]. In 2014, RIPP put the HPPO technology into a 100 kt a^−1^ industrial demonstration. In 2020, the HPPO technology package with a production capacity of 300 kt a^−1^ passed technical appraisal.

### Alternative methods for PO production

#### Direct epoxidation with O_2_

**Scheme 3.**
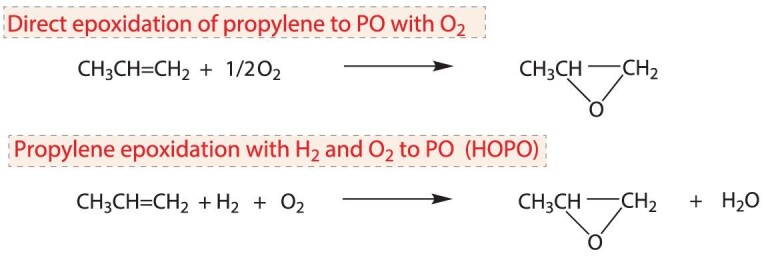
Alternative methods for the production of PO.

Direct epoxidation of propylene with O_2_ is known as the ‘dream reaction’ for the synthesis of PO (Scheme 3) [65], which can occur in the liquid or gas phase. In the liquid phase, moderate conversion and selectivity were obtained by using Mo-, W- and V-based catalysts [65]. In the gas phase, the catalysts are mainly based on active components including Ag [66], Cu [67] or TiO_2_ [68]. There are also MoO_3_- and Bi-based catalysts [69] and photocatalysts [70]. Unfortunately, the high reaction temperature or the unsatisfactory conversion/selectivity over the existing catalysts necessitates the development of more efficient catalyst systems for direct epoxidation of propylene with O_2_.

Direct photocatalytic propylene epoxidation using O_2_ has also been reported for the production of PO. Although numerous efforts have been made, this approach suffers from unsatisfactory PO yield (low propylene conversion and PO selectivity) and low turnover frequency (TOF) for industrial production, aside from the possible deactivation of the photocatalyst. Therefore, the mechanism of the photo-epoxidation of propylene should be elucidated and the kinetics should be determined for the rational design of effective photocatalysts [71].

Kube *et al*. [72] discovered that when propane is oxidized at elevated temperature over apparently inert materials such as BN and SiO_2_, not only propylene but also appreciable amounts of PO are generated, along with only small amounts of CO_2_, which represents an environmentally friendly route towards the production of propylene and PO in one step. An attractive feature of this reaction is that complex catalyst development is not necessary. However, this new process requires high temperature and uses expensive He–O_2_ mixture as the feed gas, and propylene rather than PO prevails in the product. Currently, process optimization should be focused on the utilization of renewable energy or the development of efficient catalysts to lower the reaction temperature and increase the use of inexpensive air as the feed gas.

#### Epoxidation with H_2_ and O_2_

As compared to direct epoxidation with O_2_, propylene epoxidation with H_2_ and O_2_ to PO (HOPO) is more active and selective (Scheme 3). Since noble metals, including Pt, Pd, Au and Ag, are efficient catalysts for the production of H_2_O_2_ or active oxygen species from H_2_ and O_2_, and TS-1 zeolite is effective in catalyzing propylene oxidation to PO by H_2_O_2_, the active HOPO catalysts are mainly TS-1 zeolite- or Ti-containing oxide-supported noble metals [73–75]. However, the Pt- and Pd-based catalyst systems have drawbacks such as low hydrogen utilization due to catalytic decomposition of H_2_O_2_ by Pd and Pt and fast catalyst deactivation, while the low PO selectivity at high conversion and poor stability restrict the application of the Ag/TS-1 catalyst.

Since Haruta and co-workers first discovered that the Au NPs supported on anatase TiO_2_ could convert propylene to PO via HOPO [75], Au-Ti catalysts have received a lot of attention due to their high PO selectivity at high propylene conversion and high hydrogen utilization. Based on a rough estimation for the commercialization of the HOPO method, Sinha *et al*. [76] set the research targets as: one-pass propylene conversion of 10%, PO selectivity of 90% and hydrogen utilization of 50%.

The HOPO reaction follows a bifunctional catalytic mechanism over the Au-Ti catalysts. Bravo-Suárez *et al*. [77] suggested that H_2_ and O_2_ generated H_2_O_2_ on Au first, which then transferred to neighboring Ti to produce the Ti-OOH species for the oxidation of propylene adsorbed on Ti. Qi *et al*. [78] found that Au/Ti-SiO_2_ exhibited better activity and stability than Au/TiO_2_, mainly attributed to the presence of isolated Ti^4+^ sites on the Ti-SiO_2_ support. The isolated Ti^4+^ sites play an important role in the selective deposition of Au particles and the selective production of PO, which is also held for other Ti-containing siliceous zeolite-supported Au catalysts.

However, the isolated Ti^4+^ sites cause ring-opening of PO to form the bi-coordinated propoxyl species [77], which blocks the micropores, making the Au in the pores unavailable for catalysis [79]. Based on this deactivation mechanism, Feng *et al*. [79,80] proposed two strategies for preparing stable catalysts. One is the use of uncalcined TS-1 zeolite as the support, with the micropores being blocked by the template (TS-1-B). In this way, the Au NPs were selectively loaded on the exterior of TS-1-B. The production rate of PO was maintained at 127 g_PO_ kg_cat_^−1^ h^−1^ for 30 h [79]. The other is the preparation of mesoporous TS-1 zeolites (MTS-1 [80], HTS [81] and TS-1/S-1 [82]) as the supports. Au/MTS-1 showed excellent PO selectivity of 95% and retained a high PO production rate of ^−^42 g_PO_ kg_cat_^−1^ h^−1^ over 40 h. The high diffusion ability and hydrophobicity of MTS-1 facilitated the diffusion and desorption of PO, which improved the PO selectivity and inhibited the ring-opening of PO for coke formation.

## EPICHLOROHYDRIN PRODUCTION

### Current status

Among epoxy compounds, the production of ECH ranks third after ethylene oxide and PO. ECH has wide uses and is an important raw material mainly for the synthesis of epoxy resins. ECH is also used for the production of solvents, fiberglass, nitroglycerine explosives, adhesives, coatings, ion exchange resins, electrical insulation products, plasticizers, flame retardants, paper wet strength agents, surfactants, chemical stabilizers, chemical dyes and pharmaceuticals. Moreover, ECH can be used as a solvent for cellulose resins and cellulose ethers and for water treatment [83]. In 2021, the global production capacity of ECH exceeded 3000 kt a^−1^, of which China accounted for ∼50%.

**Scheme 4.**
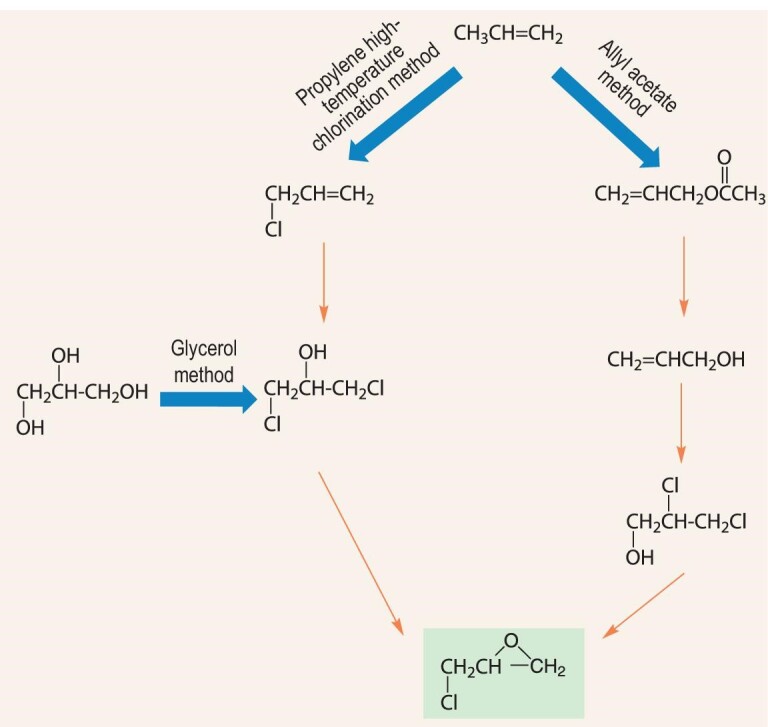
The commercialized methods for ECH production.

At present, the commercialized methods of ECH production are mainly based on the propylene high-temperature chlorination method, the allyl acetate method and the glycerol method [84,85] (Scheme 4). Since these methods all involve 1,3-dichloro-2-propanol (1,3-DCP) generation and saponification to ECH, they can be collectively classified as the 1,3-DCP saponification method. During chlorination, the equipment corrosion is serious, and 1,3-DCP saponification produces a large amount of wastewater and waste residue. For the production of 1 t of ECH, at least 0.6 t of CaCl_2_ are produced, the Cl atom utilization is only 25%, and the total atom utilization is only 50%. In 2019 China put forward restrictions on saponification units for ECH production, so the market is in dire need of new green synthesis technology for ECH.

### Epoxidation of 3-chloropropylene to epichlorohydrin

**Scheme 5.**
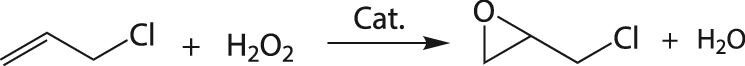
Direct epoxidation of 3-chloropropylene to ECH with H_2_O_2_.

The direct epoxidation of 3-chloropropylene to ECH with H_2_O_2_ (Scheme 5) does not involve the chlorination and saponification steps, thus avoiding the use of HClO and generation of CaCl_2_. As a result, equipment corrosion and solid waste discharge can be significantly reduced. Therefore, a number of research institutes have studied the production of ECH by direct epoxidation of 3-chloropropylene, focusing on catalyst screening and modification [86], solvent composition [87], product purification, reaction kinetics and reactor [88]. Degussa, DOW Chemical and BASF have patent layouts on direct epoxidation of 3-chloropropylene to ECH, but no subsequent pilot or industrial demonstration has been reported. The reasons mainly lie in the unsatisfactory catalyst lifetime, complicated catalyst separation procedure, and difficulty in separating ECH from the solvent.

### 3-Chloropropylene epoxidation technology of RIPP

Based on laboratory-scale studies on continuous operation of the stirred autoclave–membrane separation coupling process for epoxidation of 3-chloropropylene to ECH, RIPP identified the following key problems to be solved: (i) the TS-1 zeolite deactivates quickly, resulting in a short single-pass lifetime; (ii) the catalyst is regenerated by high-temperature solvent washing outside of the reactor and calcination, which is complex and labor-intensive; and (iii) the epoxidation product is difficult to separate from the methanol solvent by direct distillation, and ECH will undergo ring-opening reactions in the presence of methanol and water during distillation separation, which significantly reduces the yield of ECH.

To deal with these challenges, the formulation of the catalyst was optimized to improve stability. A new tubular fixed-bed process was developed to solve the technical difficulties brought about by the direct use of the TS-1 zeolite raw powders, such as separating the catalyst from the liquid product and the complicated catalyst regeneration process. Taking advantage of the strong heat extraction characteristics of the tubular reactor, stable control of the reaction temperature was achieved. The fixed-bed continuous epoxidation process was realized on the basis of catalyst regeneration using the novel reaction solvent near-critical online washing method. Efficient and low-energy separation of methanol and ECH was achieved on the basis of an extraction separation technology [89].

In light of above improvements, a 9 t a^−1^ pilot plant was designed and established, on which fixed-bed epoxidation reaction, extraction separation, distillation separation of the extracted products, evaluation of catalyst lifetime, and online catalyst regeneration were accomplished. The average H_2_O_2_ conversion was 99.0%, and the ECH yield based on 3-chloropropylene was 94.2%. The quality of the ECH product meets the highest national standard for epoxy resin production. The cumulative catalyst lifetime was 3804 h [90].

Starting from 2017, RIPP in collaboration with other institutes carried out supplementary tests on: (i) the single-pass catalyst lifetime in large-diameter tubular reactors and the extraction separation of the epoxidation products; (ii) single-pass catalyst lifetime using 30% H_2_O_2_ for epoxidation, extraction separation of epoxidation products, distillation separation of the aqueous phase of the extraction, and distillation separation of the extracted 3-chloropropylene phase; and (iii) catalytic decomposition of residual H_2_O_2_ in methanol aqueous solution. On the basis of pilot studies, a process composed of a fixed-bed epoxidation reaction, two-column continuous extraction and high-efficiency separation, solvent double-effect recovery, product separation, and catalyst online regeneration was developed, and a 50 kt a^−1^ ECH process package was developed for the first time. As compared to the traditional methods, it is green and clean with lower 3-chloropropylene consumption and greatly reduced waste emissions.

## CYCLOHEXANONE AMMOXIMATION TO CYCLOHEXANONE OXIME

### Current status

**Figure 6.**
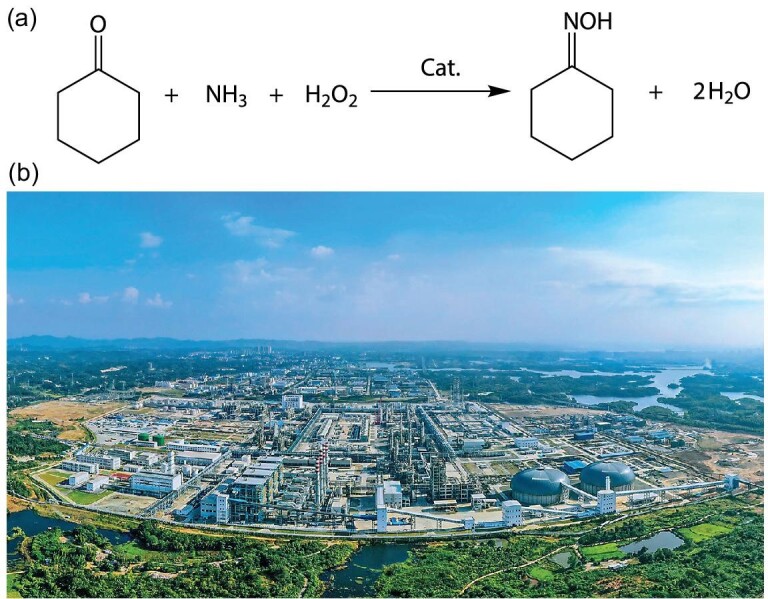
(a) One-step synthesis of CHO using H_2_O_2_ as the oxidant and NH_3_ as the nitrogen source. (b) The 300 kt a^−1^ CHO production unit based on RIPP's technology.

Caprolactam (CPL), as the monomer for nylon-6 and engineering plastics, is widely used in textile, automobile, electronics and other industries. Global CPL production exceeded 6000 kt, and ∼90% was produced by the rearrangement of CHO [91]. The production of CPL is technically complicated, involving multiple reactions and subsequently multi-step refining processes. As the key step for CPL production, the production of CHO traditionally uses the hydroxylamine method, which involves NH_3_ oxidation, N_2_O reduction to hydroxylamine, cyclohexanone ammoximation by hydroxylamine to CHO, and ammonium decomposition [92]. As the NH_3_ oxidation reaction emits NO*_x_*, the NH_3_ utilization is only 60%, which is the main source of NO*_x_* in the tail gas of the CPL plant. Meanwhile, to maintain the pH of the reaction solution at 7 by means of the hydroxylamine method, NH_3_ is used to neutralize H_2_SO_4_ dissociated from hydroxylamine sulphate, which leads to the generation of 2.5–2.7 t of (NH_4_)_2_SO_4_ per 1 t of CHO. In the 1980s, Enichem achieved one-step highly selective synthesis of CHO by the *in-situ*-formed reactive hydroxylamine using H_2_O_2_ as the oxidant, the TS zeolite as the catalyst and NH_3_ as the nitrogen source (Fig. 6a). As hydroxylamine is generated and consumed *in situ*, this method does not demand the consumption of SO_2_ to stabilize hydroxylamine and consequently no (NH_4_)_2_SO_4_ is generated. One-step ammoximation of cyclohexanone by H_2_O_2_ to CHO is simple, mild and clean, and has high nitrogen utilization.

To solve the problems of unstable activity and selectivity of the TS zeolite, poor preparation reproducibility and undesired decomposition of H_2_O_2_ caused by extra-framework Ti species, RIPP developed a new hydrothermal synthesis–secondary structural modification strategy for the synthesis of micro-sized hollow TS zeolite, which reduced the extra-framework Ti species and hence the degradation of H_2_O_2_, and improved the utilization of H_2_O_2_, the activity of the TS zeolite and the reproducibility of the preparation. To impede the inclination towards dissolution and loss of the TS zeolite during the reaction, the deactivation mechanism and the regeneration of the TS zeolite were studied, which revealed that the deactivation is caused by the dissolution and loss of framework Si species. Hence, the stability of the TS zeolite was improved by adding silica-containing additives in the reaction medium [93].

To improve the mass transfer among H_2_O_2_, cyclohexanone, NH_3_ and TS zeolite, raw hollow TS zeolite powders (particle size of 0.2 μm) were directly used as the catalyst. Combined with high-efficiency membrane separation technology, a slurry-bed–membrane separation process was used to realize the uninterrupted separation and recycling of the microscale TS zeolite. Furthermore, the problem of membrane fouling by the zeolite in an alkaline reaction system was solved, and long-term continuous and stable operation of the membrane separation process in large-scale industrial production was realized [94].

The process for continuous cyclohexanone ammoximation using H_2_O_2_ based on TS zeolite raw powder−single autoclave slurry-bed−membrane separation gave cyclohexanone conversion of ≥99.9%, CHO selectivity of ≥99.5%, and a utilization of H_2_O_2_ and NH_3_ of 90%–100%. As compared to the cyclohexanone ammoximation process using hydroxylamine salt, the nitrogen utilization is increased from 60% to >85% with no NO*_x_* emissions, the reaction process is simplified, and the plant investment is reduced [95]. Recently, a 300 kt a^−1^ CHO production unit based on this technology was successfully established (Fig. 6b).

### New progress in cyclohexanone ammoximation

Pickering interfacial catalysis without the addition of solvents is regarded as an important bi-phase green catalytic system, which has good environmental and economic benefits. This novel approach combines the merits of heterogeneous catalysis and phase transfer with increased interfacial contact area, enhanced interphase mass transfer, effective product separation and simplified recovery of heterogeneous catalysts [96]. Cyclohexanone ammoximation with H_2_O_2_ using the TS-1 zeolite is a biphasic catalytic system that requires the addition of solvents to increase the immiscibility of the reactants, which complicates the reaction process and requires additional purification steps for solvent recycling. It is highly desirable to design a Pickering interfacial catalysis system for this reaction. Lv *et al*. [97] synthesized hydrophobic hollow TS-1 zeolite particles by post-desilication accompanied by pre-crystallization. With these zeolite particles as the emulsifier, stable Pickering emulsion of cyclohexanone–H_2_O was prepared. In cyclohexanone ammoximation, the HTS-1–393(2)-S zeolite gave the activity of 247.3 mol mol_Ti_^−1^ h^−1^ and showed good recyclability. Pre-crystallization reduced the average emulsion droplet size, which is beneficial to interphase mass transfer.

The synthesis of TS zeolites requires expensive organic structure-directing agents (e.g. tetraalkylammonium hydroxides and piperidine). In industrial cyclohexanone ammoximation, the use of *t*-butanol solvent to change the reaction system from liquid−liquid−solid to liquid−solid for much better reaction performance is not environmentally benign and greatly increases the reactor volume and the downstream separation cost. In addition, the separation and recovery of the small TS zeolite particles is challenging. Hence, the development of an alternative catalyst for organic solvent-free cyclohexanone ammoximation is attractive. Wang *et al*. [98] synthesized a nanowire-composed urchin-like Nb_2_O_5_ catalyst. In cyclohexanone ammoximation in pure water, this catalyst afforded cyclohexanone conversion of 98.0% and CHO selectivity of 88.9%, which was attributed to its large surface area, high Lewis acid density, strong acidity and the unique nanostructure.

By introducing a porous ceramic membrane distributor in the reactor for the microscale distribution of H_2_O_2_, Mao *et al*. [99] developed a new organic solvent-free cyclohexanone ammoximation process. During the reaction, the membrane distributor produced tiny H_2_O_2_ droplets, which enhanced the mixing of the aqueous phase with the organic phase and improved the selectivity to CHO. Under optimal operation conditions, the cyclohexanone conversion was ca. 99.5% and the selectivity to CHO was ca. 100%.

Microreactors are effective for providing fast mass transfer and high mixing performance. To realize an economic and green process, Hu *et al*. [100] developed a T-junction microreactor system in ultrasonic field for liquid−liquid−solid cyclohexanone ammoximation. The application of two T-junction micromixers ensured not only a rapid oxidation reaction between NH_3_ and H_2_O_2_ on the TS-1 zeolite, but also an efficient interphase mass transfer to satisfy the needs of instantaneous reaction between hydroxylamine and cyclohexanone. Under optimal reaction conditions, the cyclohexanone conversion was 99.87% within 3 min as compared to 80 min on the slurry-bed reactor. Since *t*-butanol was not used, the organic solvent recovery section was reduced, which simplified the process and reduced the production cost. Besides, the catalytic efficiency of the TS-1 zeolite was improved more than 7-fold. This work shows promise for the environmentally friendly and economic production of CHO by reactor engineering.

Although industrial cyclohexanone ammoximation processes based on H_2_O_2_/TS-1 have excellent catalytic selectivity [95], excessive H_2_O_2_ is typically required due to its low stability under high temperatures and high pH reaction conditions. In addition, the pre-formed H_2_O_2_ needs to be transported from a centralized production site where it is produced in concentrations much higher than those required for the ammoximation reaction. The required dilution wastes the energy previously used in the distillation and concentration steps. In addition, due to the instability of H_2_O_2_, acid and halide stabilizers are added to prevent it from degradation during transport and storage, which in turn affects catalyst stability, reduces reactor life due to corrosion, and incurs significant costs associated with their removal from the product [101].

**Scheme 6.**
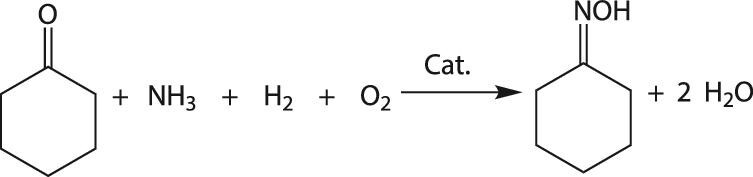
One-step synthesis of CHO via *in-situ* H_2_O_2_ synthesis.

Hutchings and co-workers [102] demonstrated that by supporting AuPd alloy NPs on TS-1 zeolite as the bifunctional catalyst, it is possible to generate H_2_O_2_  *in situ* for CHO production (Scheme 6) with a selectivity of >95%, which is comparable to the current industrial route. The alloying of Pd with Au improved catalytic activity. Although the H_2_O_2_ synthesis rate was higher over the Pd catalyst, the modification with Au inhibited the competitive H_2_O_2_ degradation pathways, which enhanced H_2_ utilization and promoted the release of the oxidant from the metal surface for utilization by the TS-1 component. It is worth noting that the formation of the PdAu nanoalloy was also conducive to improving the stability of the catalyst [103]. The introduction of low contents of Pt into the AuPd NPs further promoted the *in-situ* synthesis of H_2_O_2_ and hence the production of CHO [104]. Technoeconomic evaluation estimated 13% savings on material costs alone for the *in-situ* approach as compared to the industrial process. This approach eliminates the need to transport and store high-concentration and stabilized H_2_O_2_, which may have substantial environmental and economic benefits. As such, the *in-situ* route has the potential to supersede the current industrial route to CHO [101].

## CONCLUSIONS AND OUTLOOK

With more than two decades of endeavor, RIPP has mastered proprietary slurry-bed technology for H_2_O_2_ production, which strongly supports the development of green oxidation/ammoximation technologies in PO, ECH and caprolactam industries and creates significant economic and social benefits. Among them, caprolactam green production technology with cyclohexanone ammoximation as the core increases the self-sufficiency rate of caprolactam in China from 15% to 98%, transforming China from being import dependent to being the world's top caprolactam producer. New industries on the scale of ¥500 billion are thus formed, which can promote downstream industries on the scale of trillions of yuan. In 2020, the caprolactam green production technology won the China Industry Award. The Ministry of Industry and Information Technology and the Ministry of Ecology and Environment established Hunan Petrochemical's off-site construction project supported by the caprolactam green production technology as a benchmark for the relocation of hazardous chemical production enterprises along the Yangtze River, as it contributes greatly to the protection of clean water. The mastery of HPPO technology means that SINOPEC is the third patentee in the world, which breaks the monopoly of foreign technology. However, except for the lengthy process flow for the anthraquinone process, the use of heavy aromatics and esters as the solvent, the unavoidable degradation of expensive anthraquinone, and the requirement of H_2_ and O_2_ necessitate the development of a more affordable, clean and safe H_2_O_2_ production method.

The advantage of H_2_O_2_ synthesis by means of H_2_/O_2_ fuel cells is that it is capable of producing H_2_O_2_ at a high rate and high concentration in a safe and green manner. This electrocatalytic method has a better chance of being the next-generation H_2_O_2_ synthesis technology than the H_2_–O_2_ direct synthesis method and photocatalytic method in terms of safety and efficiency. However, the preparation of highly active catalytic materials for the electrode that synergize the selectivity of the cathodic catalytic reaction with the interaction of the three-phase interfaces of O_2_ (gas phase), electrocatalyst (solid phase) and electrolyte (liquid phase) needs to be studied in depth to improve the current efficiency and the concentration of H_2_O_2_. And moving the process from basic research to a viable technology requires a lot of research work. The future research focus is not only on the electrocatalyst itself, but also on how to optimize the diffusion across the electrode by means of regulating the microenvironment of the electrocatalytic surface (e.g. porosity, roughness and wettability) [105] and optimizing the gas flow channel of the flow field plate of the gas diffusion electrode through modeling and experimentation [46].

The HPPO process requires high H_2_O_2_ concentration, which leads to a high decomposition rate, making it difficult to transport over long distances. Hence, the HPPO process has to be accompanied by a local H_2_O_2_ production unit, which greatly increases the investment. In addition, the process requires methanol as the solvent, which requires a huge amount of steam for separation and purification, thus increasing the energy consumption.

The HOPO process overcomes the difficulty of long-distance transport of high-concentration H_2_O_2_ and does not require additional H_2_O_2_ production equipment, so it is considered the most promising alternative to the HPPO process. However, the feedstock contains flammable gases (C_3_H_6_ and H_2_) and oxidizing gas O_2_. Aside from H_2_, the explosion range of C_3_H_6_ in O_2_ is also very broad (2.0%–59%), so there is great explosion risk during the pre-mixing and reaction. Due to a lack of experimental explosion limit data and an explosion limit prediction method suitable for a system containing a variety of flammable gases, a volume ratio of C_3_H_6_/H_2_/O_2_/N_2_ of 1/1/1/7 is often used during the study, which results in low catalytic performance and PO outlet concentration that cannot meet the requirement for commercialization. To avoid explosion risk, a microchannel reactor [106], membrane reactor with separate feed of H_2_ [107], catalytic membrane reactors [108] and a porous permeation membrane reactor [109] have been proposed for this reaction. However, the performance of these reactors is limited by the low concentration of H_2_ or O_2_. Therefore, the catalytic reaction kinetics, either experimental or theoretical [110], reactor conceptual design and optimization, and explosion limit prediction need to be further studied. In addition, the products from HOPO are more complex than the chlorohydrin and co-oxidation methods, and the related separation process has not yet been established, so the design of a PO separation and purification process to match this method is of great importance. Moreover, it is still necessary to further improve the single-pass stability and activity of the catalyst on the basis of comprehensive studies on coking kinetics, deactivation kinetics, and screening and optimization of the promoters.

The TS-1 zeolite/H_2_O_2_ catalytic system is used to synthesize ECH by direct epoxidation of 3-chloropropylene, which shortens the process flow and also reduces the amount of chlorine gas. This process is still affected by the high preparation cost and low activity and stability of the catalyst, the low product yield, and solvent interference. Therefore, further optimization of the catalyst preparation method and the development of a method to eliminate solvent interference are necessary.

For cyclohexanone ammoximation using *in-situ*-formed H_2_O_2_, future research work should be focused on the iterative development of both active sites, i.e. the Ti^4+^ center within the TS-1 zeolite responsible for hydroxylamine synthesis, and the metal species responsible for H_2_O_2_ production. It is apparent that there is also a need to improve the reactivity and H_2_ utilization of the catalyst, while diffusion limitations associated with the TS-1 pore structure should also be considered. Furthermore, it is known that the loss of TS-1 crystallinity and the formation of surface TiO_2_ species due to the presence of ammonia in the reaction stream can lead to catalyst deactivation in industrial use and promote the dissolution of noble metals. Moreover, there is still a need to assess the long-term stability of the immobilized noble metal species under real reaction conditions and over extended reaction time [103].
